# Management of Pediatric Strokes With Alteplase (Tissue Plasminogen Activator)

**DOI:** 10.7759/cureus.17088

**Published:** 2021-08-11

**Authors:** Asia Filatov, Jaime Alvarez, Jessica Seibert, Marc Swerdloff

**Affiliations:** 1 Neurology, Florida Atlantic University, Boca Raton, USA; 2 Emergency Medicine, Boca Raton Regional Hospital, Boca Raton, USA; 3 Neurology, Boca Raton Regional Hospital, Boca Raton, USA; 4 Neurology, Marcus Neuroscience Institute, Boca Raton, USA

**Keywords:** pediatric stroke, alteplase, stroke, tissue plasminogen activator (tpa), tpa

## Abstract

In the pediatric population, ischemic stroke is rare. Pediatric patients with acute ischemic stroke are eligible for intravenous thrombolysis and/or mechanical thrombectomy. However, due to the rare occurrence of strokes and national shortage of pediatric neurologists, timely diagnosis and appropriate treatment can be challenging. We report a case of a 16-year-old female who presented with an acute ischemic stroke to our adult comprehensive stroke program.

## Introduction

Ischemic stroke in the pediatric population is rare with an incidence ranging from 2.3 to 13 per 100,000 children [1]. However, stroke in children in the acute setting can be missed because of its low prevalence. The rare nature of stroke in children emphasizes the need for multidisciplinary alliance and approach to diagnosis and treatment. Past attempts to study the safety and efficacy of alteplase in children via a controlled trial were unsuccessful secondary to the medical comorbidities, with the population being vulnerable and diagnostic difficulties associated with pediatric stroke. Treatment varies by institution and should be addressed on a case-by-case basis. The use of intravenous tissue plasminogen activator is feasible and safe in children. The objective of this case report is to highlight the necessity for the development of a standard procedural policy to address stroke in the pediatric population.

## Case presentation

**Figure 1:**
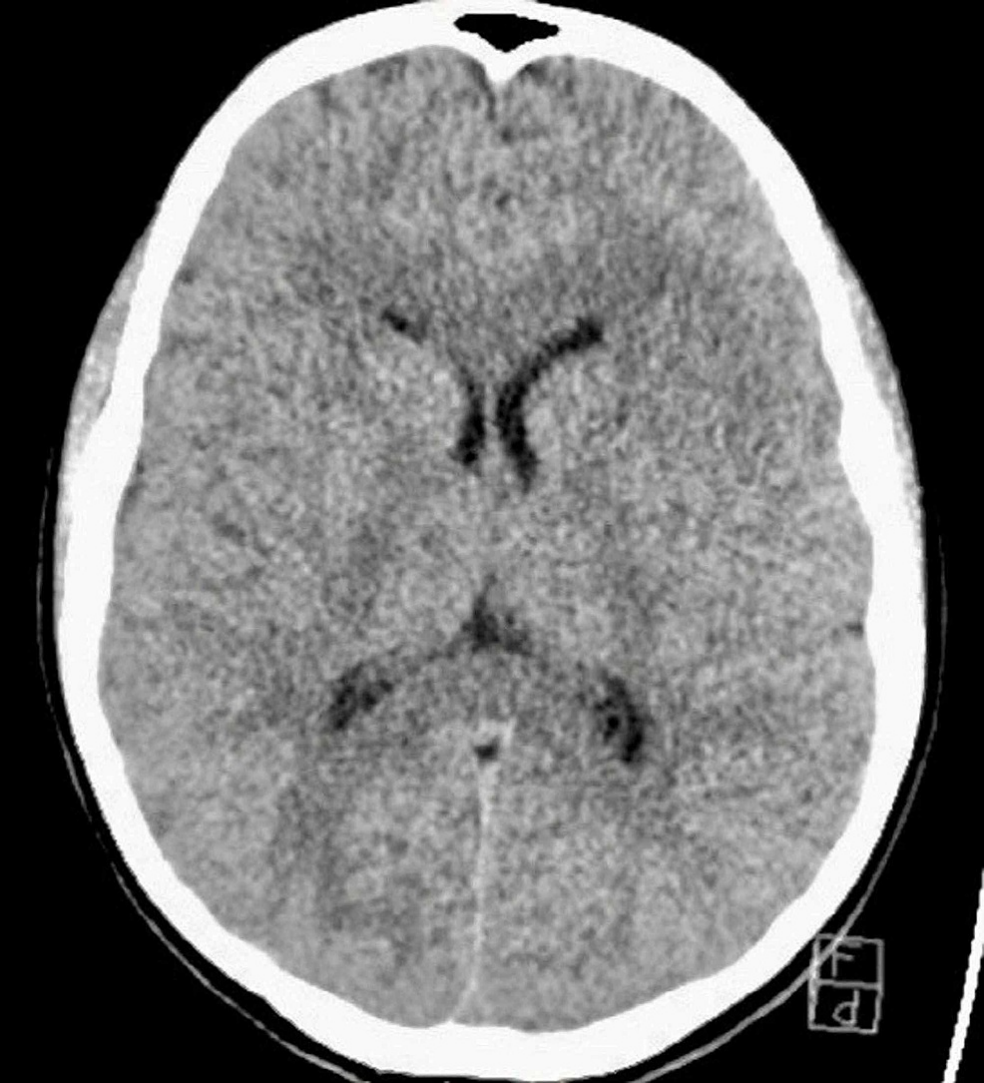
CT head: normal

**Figure 2:**
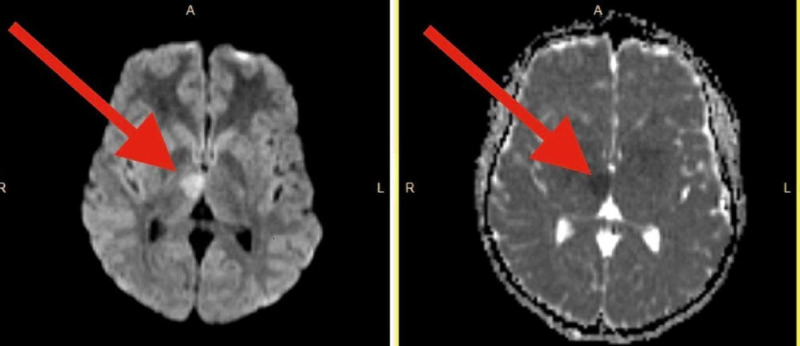
Brain MRI: right thalamic infarct with a small penumbra on diffusion weighted imaging and apparent diffusion coefficient mapping

A 16-year-old female volleyball player presented to the emergency department with left facial weakness, slurred speech, horizontal nystagmus and left arm weakness. Her last known normal was 45 minutes prior to arrival. She was playing laser tag when she bumped heads with her friend in the course of the game followed by the onset of her symptoms. Emergency Medical Services (EMS) were notified and the patient was transferred to the nearest comprehensive stroke center. The patient was not taking any prescribed medications and was not on oral contraceptives. She had no chronic medical conditions and denied regular use of any medication including oral contraceptives. On arrival, her blood pressure was 117/71 mmHg, blood glucose was 103 mg/dL and the National Institutes of Health Stroke Scale score was 6 for left facial droop, dysarthria, expressive aphasia and left arm (3-/5) and left leg (4/5) weakness. In addition, she had un-sustained left gaze-evoked nystagmus, left skew deviation, but was able to follow commands. A stat CT of the head was normal (Figure 1). Due to the uncertainty of a possible stroke, a stat MRI brain was obtained and showed a right thalamic infarct with a small penumbra on diffusion/perfusion imaging (Figure 2). Telemedicine declined to see the pediatric patient. The adult neurologist on call was not credentialed to evaluate the pediatric case nor had the appropriate training. Further delay occurred in the process of trying to reach the nearest pediatric facility. When discussed with the pediatric hospital, they were hesitant on administering alteplase and ultimately recommended not to give alteplase. Subsequent deliberations took place among the onsite emergency room physician, on-call physician, stroke neurologist, and the Chief of Neurology. A consensus was reached after discussion with the patient’s parents to administer IV alteplase using our adult stroke protocol and lastly, a decision was made to transfer the patient to a pediatric facility. The door-to-needle time was 1 hour and 45 minutes. Alteplase administration was ultimately the correct decision with complete resolution of her symptoms. When the patient was transferred to a pediatric hospital after 24-hour alteplase protocol to monitor for hemorrhagic conversion, she was found to have a large patent foramen ovale. Repairing the congenital heart defect reduces the risk of subsequent stroke. The patient was evaluated by cardiothoracic surgery and underwent corrective surgery.

## Discussion

This case report highlights the effectiveness of thrombolysis in a pediatric population. Early recognition of stroke symptoms can lead to children arriving to the hospital within the recommended 4.5-hour time frame and then intravenous alteplase can be administered [2]. The current standard of care dictates the administration of alteplase based only upon unenhanced CT of head; advanced imaging is not required to stratify patients. Further imaging modalities include CT angiography and CT perfusion to evaluate for large vessel occlusion and possible thrombectomy. Definitive evidence on advance imaging allows for the identification of penumbra, an area at risk, which is the target of stroke intervention such as thrombectomy.

IV alteplase is given at the same dose of 0.9 mg/kg (maximum dose of 90 mg) to pediatric patients (age 2-17 years) as in adults. Alteplase should be given within 4.5 hours of symptom presentation [3]. Ten percent of the total dose is to be administered as an IV bolus over the span of 1 minute and the remaining 90% of the dose is infused over the span of 60 minutes [4].

The World Health Organization stroke guidelines recommend the use of thrombolytic agents or clot-dissolving drugs, such as alteplase, for restoring blood flow when given rapidly enough to help reduce brain damage, in the pediatric population [5]. Alteplase acts by binding to fibrin at lysine-binding sites resulting in the conversion of plasminogen to plasmin [6]. The effectiveness of alteplase is shown in multiple trials and is approved by the FDA for patients over the age of 18 [7]. The agent may be used in children aged 2-17 years when the time of onset of arterial ischemic stroke is known. It should not be used if the time since the onset of stroke symptoms is greater than 4.5 hours or unknown. Older adolescents may meet standard adult guidelines for the administration of alteplase [1]. Current guidelines dictate that children age 2-17 years must have a proven occlusion on CT angiogram or MR angiogram with corresponding stroke on MRI diffusion-weighted imaging (hemiplegia with normal CT is not sufficient). All inclusion criteria must be met and exclusions criteria must be applied. Thrombolytic therapy is not recommended unless the diagnosis is established by a physician who has expertise in the diagnosis of pediatric stroke, and the appropriate neuroimaging has been done and assessed by physicians who have expertise in reading this imaging study. If CT or MRI demonstrates changes of a recent major/extensive infarction, such as sulcal effacement, mass effect, edema, or possible hemorrhage, then thrombolytic therapy should be avoided. The risk of hemorrhage in children is 5%. Symptomatic hemorrhage and asymptomatic hemorrhage occurs in approximately 5%-6%, similar to that in adults [8]. IV alteplase is beneficial when administered for managing acute ischemic stroke symptoms; its benefit is uncertain however, if administered more than 4.5 hours after the onset of symptoms [9].

Lehman and colleagues affirmed that the use of alteplase in children is associated with no treatment complications; however, concern has been raised by other studies [4]. There is a lack of safety data concerning the use of alteplase in children. The Thrombolysis in Pediatric Stroke study attempted to evaluate the use of alteplase in children; however, the enrollment of participants was scarce leading to insufficient data collection and study results [10].

Treatment varies by institution and the use of alteplase should be considered in children with high suspicion for stroke [11].

Alteplase administration in children is associated with the risks similar to those observed in adults. Common adverse effects of alteplase therapy include anaphylaxis, angioedema, fever, and bleeding. Other effects such as cholesterol embolization have been reported [12]. Alteplase may increase the risk of intracranial hemorrhage in children, in which occurrence may be increased by the size of an infarct similar to that in the adult population. There is evidence that the thrombolytic activity of alteplase is associated with increased rates of mortality and morbidity, perhaps due to the increased rate of symptomatic hemorrhage [10,13].

## Conclusions

Our case report shows the safety and efficacy of alteplase when appropriately administered in a pediatric patient with acute ischemic stroke. It also illustrates the need for evidence-based algorithms for acute stroke care in the pediatric population. Our patient adds to the body of evidence that IV alteplase is a safe and effective treatment for stroke in children. “Time is brain” when giving IV alteplase to stroke patients when there are signs of tissue damage for stroke on neuroimaging. There is a lack of clarity and consistency across institutions regarding pediatric guidelines for alteplase administration, especially in pediatric patients younger than 18 years of age. However, there is evidence that alteplase has beneficial outcomes when used in pediatric patients (age 2-17 years) for the management of acute ischemic stroke.
